# Addressing individual needs in mindful eating: a latent profile analysis and exploration of demographics and social-cognitive beliefs

**DOI:** 10.1080/21642850.2025.2519587

**Published:** 2025-06-30

**Authors:** Christian E. Preissner, Dennis de Ruijter, Anke Oenema, Hein de Vries

**Affiliations:** a Department of Health Promotion, Maastricht University Maastricht, Netherlands; b Department of Health Psychology, Open Universiteit, Heerlen, Netherlands

**Keywords:** Mindful eating, determinants, social-cognitive, I-Change Model, profiles

## Abstract

**Introduction:**

To promote mindful eating it may be relevant to take different eating profiles into account. This prospective study aimed to (i) identify the existence of potential respondent subgroups regarding mindful eating and (ii) compare these profiles on socio-demographic characteristics and social-cognitive beliefs about mindful eating using the I-Change Model (ICM).

**Methods:**

Dutch adults (M_age_ = 52.6; 53% male) responded to an online survey at baseline (*N* = 615) and 3-months (*n* = 513) follow-up asking about social-cognitive beliefs about practicing mindful eating based on the ICM. Following a latent profile analysis of mindful eating facets, profiles at baseline were compared on social-cognitive beliefs at follow-up using a MANOVA with Tukey-adjusted post-hoc tests.

**Results:**

Three profiles were identified (1. low awareness, high acceptance; 2. high awareness, low acceptance; 3. moderate awareness, moderate acceptance). These profiles significantly differed in their demographics and social-cognitive beliefs about mindful eating (e.g., knowledge, perceived pros and cons, self-efficacy, intention and planning to adopt mindful eating).

**Discussion:**

Findings suggest the limitations of a one-size-fits-all approach to promoting mindful eating. Interventions may need to consider different recruitment and targeted strategies based on socio-demographic characteristics and social-cognitive beliefs to ensure different groups of individuals are represented in and can benefit from interventions in a safe and accessible way.

Mindful eating has become increasingly popular in recent years as an approach to studying and targeting a variety of dietary outcomes and eating behaviors (Carrière et al., 2022; Carrière et al., 2022; Grider et al., 2021; Warren et al., 2017). Mindful eating is an event-based practice that is synchronized with food-related situations or behaviors (Mantzios, 2021; Preissner et al., 2022). Derived from Western mindfulness definitions, this behavior-specific practice consists of two fundamental mechanisms: (i) present-moment awareness (including external, environmental and internal processes) and (ii) a non-judgmental and non-reactive stance toward the awareness (Carrière et al., 2022). Together, these fundamental awareness and acceptance mechanisms are proposed to support cognitive and emotional self-regulation (Mantzios, 2023a). Despite its suggested promise (e.g. Jordan et al., 2014; Warren et al., 2017), it remains unclear for whom and why mindful eating interventions may be effective.

## Respondent differences

To enhance the understanding of mindful eating and facilitate the development of tailored interventions, it is essential to investigate the degree of homogeneity within study samples, as mindful eating skills may vary across individuals. For instance, studies using latent profile analysis (LPA) have suggested the existence of respondent subgroups in (i) mindfulness (see Lecuona et al., 2022 for a review), (ii) beliefs about mindfulness practice (Beattie et al., 2022), (iii) beliefs about behavioral outcomes (Patrick & Maggs, 2010; Stapleton et al., 2010), (iv) baseline determinants predicting treatment outcomes (Saunders et al., 2016; Tanaka & Nolan, 2018; Uckelstam et al., 2019), as well as (v) risk groups to help tailor interventions (Hillhouse et al., 2016; Rijbroek et al., 2019). Considering these findings of distinct respondent subgroups in general dispositional mindfulness, it may also be relevant to uncover latent or ‘hidden’ respondent differences in mindful eating. As adopting mindful eating behavior may be influenced by a complex array of psychological, behavioral, and environmental factors beyond sociodemographics (Preissner et al., 2022), treating a study population as a single homogeneous group may obscure meaningful differences that might explain or hinder the adoption of mindful eating strategies. For example, encouraging a person to become more conscious of homeostatic cues (e.g. hunger and satiety) may also heighten their awareness of non-homeostatic urges (e.g. eating in response to stress or emotions) (Carrière et al., 2022). Depending on an individual's dispositional level of acceptance skills, interventions that focus solely on the sensory experience of eating, without sufficiently addressing acceptance skills, may risk impairing rather than enhancing self-regulation (Kearney et al., 2012; Siemers et al., 2025). The first goal of this study is thus to identify the existence of potential mindful eating subgroups and explore their characteristics.

## Differences in behavioral determinants

Interventions encourage regularly practicing mindful eating to promote sustained behavioral practice after the end of a program (Kristeller & Jordan, 2018; Zervos et al., 2022). Nonetheless, research in the field of mindful eating has predominantly been conducted without consideration of established theoretical models that explain the adoption and maintenance of a health behavior. This is problematic, as failing to incorporate and measure constructs from established behavior change models obscures relevant participant characteristics in dispositional skills, knowledge, beliefs, motivations, and intentions that could explain (i) the sample, (ii) the adoption of mindful eating, and (ii) the effectiveness of interventions. While it is known that mindfulness practitioners tend to be Caucasian, female, middle-aged, and college-educated (Burke et al., 2017; Preissner et al., 2025a), other dispositional mindfulness-related skills and social-cognitive beliefs remain largely unmeasured in mindful eating research (Preissner et al., 2022). This creates difficulties for the effective development, targeting, and evaluation of interventions because key factors that drive behavior adoption and change are not known. Foundational determinant research is needed to identify the factors influencing mindful eating adoption.

A comprehensive model explaining the adoption and change of health behaviors is the I-Change Model (ICM; de Vries, 2017). This model integrates the ideas of the Theory of Planned Behavior (Ajzen, 1991), the Social Cognitive Theory (Bandura, 1986), the Transtheoretical Model (Prochaska & DiClemente, 1986), and the Health Belief Model (Janz & Becker, 1984). The ICM suggests that the likelihood of converting behavioral intentions into concrete actions is positively influenced by a person's ability to prepare and implement specific plans, while barriers decrease these chances. Barriers and intentions are influenced by motivational factors (i.e. attitudes, social influence, and self-efficacy beliefs). The ICM posits that these motivational factors are shaped by distal factors such as awareness (e.g. cognizance about own behavior, knowledge about the behavior, risk perceptions, cues to action), predisposing factors (e.g. psychological or behavioral factors such as personality or lifestyle), social and cultural factors (e.g. policies, economic factors), and information factors (e.g. the quality of health information, channels and sources used to obtain information). This model has previously been applied to examine a variety of eating and dietary behaviors including determinants of eating in moderation (Walthouwer et al., 2015), fruit and vegetable intake (Schulz et al., 2014), and dietary supplement use (Pajor et al., 2017).

The ICM has recently been applied in an exploratory, cross-sectional study to identify determinants of engaging in mindful eating (Preissner et al., 2022). This cross-sectional study pointed to individuals with greater behavioral cognizance (i.e. awareness of one's own actions), awareness of internal cues to eat, perceived susceptibility to developing health conditions, social support, and greater practice intentions as being most likely to engage in mindful eating. Their research further highlighted differences in social-cognitive beliefs among participants with differing levels of mindful eating practice experience (Preissner et al., 2022). That study presented an important first step in identifying determinants as well as examining key differences between different levels of mindful eating experience. The latter exploratory research aim is crucial, as examining only an overall sample conceals variations in dispositional skills, beliefs, motivations, and intentions related to behavior adoption (Beattie et al., 2022). Longitudinal studies are warranted to better understand subgroup differences in mindful eating-related characteristics and social-cognitive beliefs. The second goal of this study is thus to analyze differences in determinants of mindful eating using a prospective design.

## Methods

### Participants and sampling

We recruited a stratified sample regarding gender, education, and Dutch province from members of a nation-wide research panel (Flycatcher Internet Research BV). Respondents were representative for the Dutch population aged 18 years and older with regard to education and province (see osf.io/9ct6j). Participants were excluded if they were diagnosed with a neurodegenerative disease. Participation was voluntary and could be discontinued at any time. Informed consent was obtained from all participants. Participants were compensated for their time using reward points that could be exchanged for vouchers or gift cards, as well as a Flycatcher lottery ticket. This incentive structure was intended to facilitate the inclusion of a broad range of individuals, including those who may not have a prior interest in mindful eating. At baseline (T0), 615 individuals filled out the questionnaire. At the 3-month follow up (T1), 513 participants completed the questionnaire (83.4% retention). This study was approved by the institutional review ethics board (REC/2023/098). Further information on the sampling and an a-priori power analysis can be found in the preregistration on the Open Science Framework (OSF; https://osf.io/u9pkv).

### Measurement

#### Descriptive variables

We assessed key demographic variables, including age, gender, the highest completed level of formal education, employment status, and residential situation. In addition, we assessed height in cm and weight in kg to permit the calculation of a Body Mass Index (BMI) score. Participants could optionally disclose experienced daily limitations regarding various health conditions over the past year, including an eating disorder diagnosis (0 – ‘No‘; 1 – ‘Yes, but not limited by this‘; 2 – ‘Yes, slightly limited‘; 3 - ‘Yes, very limited‘). In addition, participants were asked to report on two items whether they had previous experience with guided meditation practice (‘yes’, ‘no’) as well as how frequently they currently engage in meditation practice (‘never’, ‘some times per year’, ‘sometimes, but not every week’, ‘regularly, but not every day’, ‘every day’). Participants were additionally presented with a lay description of ME considering both awareness and acceptance aspects. We then assessed the extent of prior experience with ME (‘never heard of it before this survey’, ‘heard about it, never tried it’, ‘some experience with it’, ‘a lot of experience with it’) and the perceived frequency of engaging in both awareness and acceptance aspects of ME (‘never’, ‘some times per year’, ‘sometimes, but not every week’, ‘regularly, but not every day’, ‘every day’); Intraclass correlation coefficient = 0.73.

#### Determinants of mindful eating behavior

Items were based on two prior cross-sectional studies (Naßenstein et al., 2022; Preissner et al., 2022) that aimed to develop a questionnaire for and investigate a range of social-cognitive beliefs associated with mindful eating in the general population. Items were developed by integrating awareness and acceptance aspects of mindful eating with previously published recommendations for the phrasing of social-cognitive beliefs (Ajzen, 1991, 2002) and previous research using the ICM (Kasten et al., 2019; Walthouwer et al., 2015).

The items from the two prior studies were reanalyzed using confirmatory factor analyses and jackknife procedures in R (maximum likelihood estimation and adjusted for non-normality) to select the most relevant items for the present study. A baseline model was run using all items. Next, one item at a time was removed from the construct, and analyses were repeated. The model fit was compared for each N-1 item solution using the comparative fit index, root mean square error of approximation, standardized root mean square residual, and Tucker–Lewis index. The item from the best fitting model was removed and analyses were repeated with one item removed until the fit indices no longer improved.

Participants were provided with a lay description of mindful eating considering both awareness and acceptance aspects before answering ICM items. We used principal component analysis for each construct separately to determine whether the corresponding items were measuring the respective component as intended. The constructs, number of items, example items, answer categories, and Cronbach's alphas are presented in Table 1. A complete overview of the ICM components and items can be found on OSF (osf.io/9ct6j).

**Table 1.** Overview of constructs and items per ICM construct.

ICM Construct	No. of items	Example items, answering points	α T0	α T1
Nutrition Cognizance	5	‘My diet adheres to the national recommendations for a healthy, balanced diet.’ *never (1) to always (6)*	.70	.66
Quality Cognizance	4	‘I am aware of why I eat (e.g. I felt hungry, food looked tempting, I felt sad, stressed or anxious).’ *never (1) to always (6)*	.79	.79
Focus Cognizance (Distractions)	6	‘When I eat, I use my mobile phone.’ *never (1) to always (6)*	.62	.65
Knowledge	7	‘Mindful eating is a form of dieting that restricts certain foods such as sugar’ *Correct (= 1), incorrect or uncertain (= 0)*	.86	.83
Cues to Action	4	‘I have seen information about mindful eating in the media (e.g. Internet, TV, newspaper).’ *strongly disagree (1) to strongly agree (7)*	.69	.72
Susceptibility	4	‘My risk of overeating is:’ *very low (1) to very high (5)*	.83	.84
Severity	4	‘For me, overeating is:’ *not very serious (1) to very serious (5)*	.81	.78
Attitude Pros	10	‘ If I eat mindfully, that would: … help me to stop eating when I am full.’ *strongly disagree (1) to strongly agree (7)*	.96	.96
Attitude Cons	10	‘If I eat mindfully, that would: … make me think too much about my food choices.’ *strongly disagree (1) to strongly agree (7)*	.86	.89
Subjective Norms	4	‘My family thinks I should eat mindfully.’ *strongly disagree (1) to strongly agree (7)*	.95	.97
Modeling	4	‘My family eats mindfully.’ *strongly disagree (1) to strongly agree (7)*	.93	.92
Social Support	4	‘My family encourages me to eat mindfully.’ *strongly disagree (1) to strongly agree (7)*	.95	.95
Self-Efficacy	8	‘How easy or difficult will it be for you to eat mindfully when you feel stressed?’ *very easy (1) to very difficult (7)*	.89	.90
Intention	3	‘For the next month, I intend to eat mindfully at least once a week.’ *strongly disagree (1) to strongly agree (7)*	.91	.92
Action Planning	7	‘I made a detailed plan to remind myself to eat mindfully (e.g. on my cell phone or Post-It notes).’ *definitely not (1) to definitely yes (5)*	.91	.91
Coping Planning (Sniehotta et al., 2005)	5	‘I made a detailed plan on what to do if something interferes with my plans to eat mindfully.’ *definitely not (1) to definitely yes (5)*	.95	.96

#### Mindful eating skills

We used a 19-item short version of the valid and reliable Dutch Four Facet Mindful Eating Scale (FFaMES; Carrière et al., 2022; Preissner et al., 2025b). The Dutch FFaMES showed the same four-factor structure as well as reliability and validity as the original *(*Preissner et al., 2025b*)*. The short-form retained the four-factor structure and showed excellent model fit (CFI = 0.99; RMSEA 90% CI [0.031, 0.040]) and similar psychometric properties (e.g. α = .80 to .95) to the original in two independent samples. The FFaMES measures participants’ non-reactance (maintaining a mental distance from immediate eating needs), non-judgment (accepting one's eating behaviors without negative self-judgment), external awareness (observing the effects of environmental factors on one's eating), and internal awareness (observing the effects of internal processes on one's eating) on a five-point Likert scale from never (1) to very often (5). Items for non-reactance and non-judgment are reverse-coded prior to calculating composite scores. Cronbach's alpha ranged between .84 (external awareness) to .95 (non-judgment) at T0 and between .85 (external awareness) and .94 (non-reactance) at T1.

### Statistical analyses

#### Descriptive statistics

Descriptive statistics using SPSS v. 28, considering a significance level of 0.05 for two-tailed analyses. Dropout analyses were conducted using logistic regression considering age, gender, education, meditation experience, and FFaMES variables. There were no missing values on FFaMES and ICM constructs, with the exception of cognizance items, for which less than 4.2% were missing. Little's MCAR test suggested that data at follow-up were missing at random (χ^2^ = 28.83, df = 27, *p* = .369). While there is no gold standard for handling missing data, it is recommended to present the results of an imputed dataset alongside the results for complete case analysis (Powney et al., 2014). The analyses described in the following using complete-case analysis were repeated using a dataset with imputations. Missing cases were imputed using the expectation maximization algorithm (Do & Batzoglou, 2008; Molenberghs & Verbeke, 2005) to ensure the least bias in estimates and power (Enders, 2001; Scheffer, 2002). The data and results using the imputed data set can be found on OSF (osf.io/9ct6j). Results after imputation showed no differences in significant variables or the directionality from the complete case analysis.

#### Latent profile analysis

One method of examining subgroups or profiles is LPA. This method is one kind of mixture model (Hancock & Samuelsen, 2008; Masyn, 2013; McLachlan & Peel, 2000; Sterba, 2013) which assumes that the patterns of means on multiple observed variables can be explained by distinct underlying latent subgroups. The present sample size exceeded recommendations for LPA (Nylund et al., 2007; Spurk et al., 2020). We conducted the LPA using the tidyLPA package in R (Rosenberg et al., 2018) to investigate the number, shape, and prevalence of specific mindful eating profiles. We followed a stepwise LPA process (Bauer, 2022; Ram & Grimm, 2009; Spurk et al., 2020), initially testing for 1 to 9 possible profiles. Model selection was guided by a variety of fit indices such as AIC, AWE, BIC, CLC, and KIC (Akogul & Erisoglu, 2017), the general interpretability of profile solutions, visual plots, as well as theoretical and content-related considerations (Bauer, 2022; Spurk et al., 2020). We hereby considered Ram and Grimm’s (2009) decision steps. A more detailed description has been provided in Preissner et al., 2025c. To enhance the robustness of our findings, all analyses were replicated at follow-up. McNemar's tests were conducted to investigate differences in profile assignment at baseline and follow-up.

#### Profile differences

Differences between profiles on socio-demographic variables were examined using frequencies, crosstabs, and analyses of variance (ANOVAs) in SPSS. A multivariate analysis of covariance (MANCOVA) with Tukey-adjusted post-hoc tests was used to examine potential differences in ICM constructs between the groups at follow-up. Age, gender, educational status, and meditation experience were entered as covariates.

## Results

### Sample characteristics

Participants were predominantly middle-aged Dutch adults with a slightly elevated BMI (see cutoffs Weir & Jan, 2025). About half of the sample identified as male. The majority of participants had completed a moderate level of education, with one fourth of the sample indicating a lower educational status. Less than a sixth indicated experience with meditation, with 6.0% meditating regularly and 1.8% meditating every day. A third of the sample indicated never having heard about mindful eating before participating in the study. Sample characteristics can be found in Table 2. The logistic regression analyses indicated that dropouts were significantly younger (*p* = .016).

**Table 2.** Sample characteristics at baseline (T0).

Variable	Total (N = 615)		Moderate Mindful Eater (n = 324)		Acceptingly Unaware (n = 154)		Non-Acceptingly Aware (n = 134)		
	M or n	SD or %	M or n	SD or %	M or n	SD or %	M or n	SD or %	F or χ2
Age	52.6	17.5	52.2	17.5	60.2	16.4	44.9	14.99	30.12*
Gender (male)	327	53.2%	184	56.8%	103	66.9%	39	29.1%	44.51*
BMI	25.9	4.8	25.5	4.17	25.5	4.47	27.3	6.27	7.41*
Education									26.27*
Low	156	25.9%	68	21.0%	61	39.6%	29	21.6%	
Moderate	255	41.5%	149	46.0%	56	36.4%	48	35.8%	
High	201	32.7%	107	33.0%	37	24.0%	57	42.5%	
Meditation Experience	94	15.3%	49	15.1%	10	6.5%	35	26.1%	21.26*
Regular Meditator	48	7.8%	29	9.0%	4	2.6%	15	9.2%	23.64*
Mindful Eating Experience									
Never heard about it	406	66.0%	205	50.5%	132	32.5%	69	17.0%	43.03*
Heard about it, never tried it	118	19.2%	68	57.6%	17	14.4%	33	28.0%	
A bit of experience	81	13.2%	47	58.0%	5	6.2%	29	35.8%	
A lot of experience	10	1.6%	5	50.0%	1	10.0%	4	40.0%	
Eating Disorder	12	2.0%	1	0.6%	1	0.7%	10	10.4%	45.29*

Note. Values may not add up to 100% due to rounding and missing values; The subgroups do not add up to 615 due to outlier removal prior to conducting the LPA; BMI = Body Mass Index; * = *p* < .01.

### Latent profile analysis (LPA)

#### Step 1: Pre-analyses

The FFaMES showed excellent fit in the CFA with a separate four-factor solution (χ2(164) = 430.36; robust RMSEA = 0.036, 90% CI [0.032, 0.04]; robust CFI = .99; robust TLI = 0.99; SRMR = 0.041). Skewness, kurtosis, and histograms revealed normal distributions (skew: −.72 to .18; kurtosis: −.61 to .78). Correlations between FFaMES facets showed moderate associations (r = −.42 to .64). Three (0.48%) and one outlier (0.19%) were identified and removed using Mahalanobis distance from T0 and T1, respectively.

#### Step 2: Latent profile analyses

We first estimated a general model for nine possible profile solutions (see Appendix A). The AIC and BIC favored a nine and seven-profile solution, respectively. The analytic hierarchy process based on the fit indices AIC, AWE, BIC, CLC, and KIC (Akogul & Erisoglu, 2017) suggested the best solution to be the model with six profiles. Careful evaluation of the fit indices, minimum group size, and visual plots indicated that (i) models with more than three profiles assigned less than 6% of the sample to the smallest group, compared to over 40% of the sample in the largest group, and (ii) did not reveal new meaningful patterns (see Appendix A). We additionally explored models with different specifications for estimating variances and covariances, such as covariances fixed to 0 or assuming equal covariances (see Rosenberg, 2021). About half of the models could not be estimated. Models assuming equal variances and equal covariances produced non-optimal sample assignments for all models (e.g. a 5% versus 95% split for two groups). Models with variances constrained to be equal and covariances fixed to 0 also produced similar results to models run using standardized variables. After careful consideration in line with Bauer (2022), Ram and Grimm (2009) as well as Spurk et al. (2020), a three-profile solution was retained (AIC = 4967.88, BIC = 5047.38, entropy = 0.75, prob_min = 0.87, prob_max = 0.90, n_min = 0.22).

#### Replication over time

The steps outlined above were repeated for the three-month follow up. The analytic hierarchy process (Akogul & Erisoglu, 2017), favored a solution estimating unequal variances and covariances fixed to 0 with four profiles. The BIC favored 3 profiles. These two models had identical entropy (0.77), with the three-profile solution showing a more favorable average class probability (min = 0.86, max = 0.90) in comparison to the four-profile solution (min = 0.73, max = 0.91), in addition to a more equal sample distribution (see Appendix A). Models with variances constrained to be equal and covariances fixed to 0 again produced similar results to models run using standardized variables. Similar to T0, the evaluation of the fit indices, minimum group sizes, and visual plots indicated more than three profiles did not reveal new meaningful groups. A three-profile solution was thus retained (AIC = 4157.80, BIC = 4234.02 0.77, prob_min = 0.85, prob_max = 0.91, n_min = 0.23, n_max = 0.52). All models can be found in Appendix A.

### Characteristics of the latent profiles

**Figure 1.**
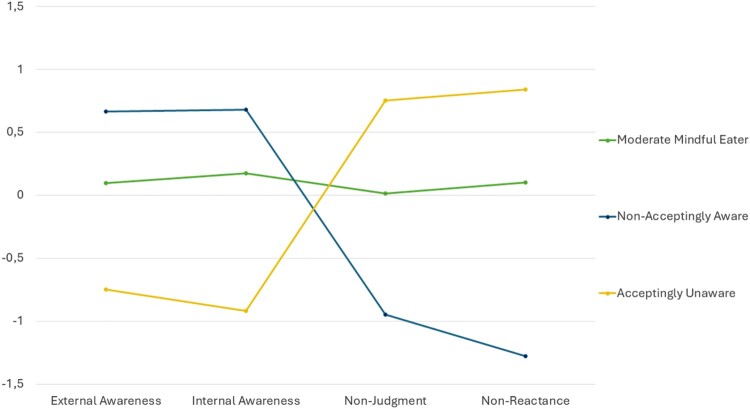
z-transformed means on FFaMES facets for the three profiles at baseline.

**Figure 2.**
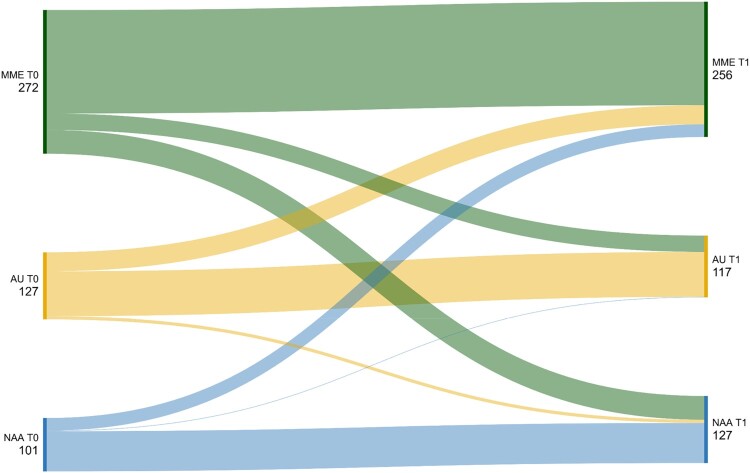
Sankey diagram showing profile allocation of FFaMES completers at T0 and T1. Note. MME = Moderate Mindful Eater; AU = Acceptingly Unaware; NAA = Non-Acceptingly Aware.

Half of the sample showed moderate mindful eating skills (n = 324, 52.9%), 25.2% (n = 154) had low awareness but high acceptance skills (‘acceptingly unaware’), and 21.9% (n = 134) had high awareness but low acceptance skills (‘non-acceptingly aware’). The three profiles at baseline with their z-transformed means per mindful eating facet are visualized in Figure 1. Proportions of the sample for those also completing the FFaMES at follow-up were similar, with 51.2% (n = 256) assigned to the group of moderate mindful eaters, 23.4% to the acceptingly unaware (n = 117), and 25.4% (n = 127) to the group of non-acceptingly aware individuals. Most participants remained in their original group from baseline to follow-up (67% to 75%). Although some participants were reassigned to another group over time, these movements were primarily to or from the group of moderate mindful eaters (MME). Using McNemar's test, we evaluated transitions between the two opposing groups: acceptingly unaware and non-acceptingly aware. The analysis revealed that the movement between these two groups was not statistically significant (*p* = .125). Specifically, 1 (1%) and 6 participants (5%) were reassigned to the opposite group over time. These LPA group assignments over time are presented in Figure 2.

Differences between profiles in demographics and meditation-related variables can be found in Table 2. All groups significantly differed from one another in mean age and BMI, with non-acceptingly aware participants being the youngest (all *p* < .001) and having the highest BMI in comparison to moderate mindful eaters (*p* < .001) and acceptingly unaware participants (*p* = .004). Non-acceptingly aware participants were predominantly female, with a higher educational status, and more prior meditation experience (see Table 2). Around 10% of this group indicated that they were limited in their daily lives due to living with an eating disorder.

### Differences in ICM constructs

The MANCOVA comparing the three profiles on ICM constructs at the 3-month follow-up (T1), controlling for demographics, was significant (F[32, 978] = 7.33, *p* < .001; Pillai's trace = .39, partial η2 = .19). Tukey-adjusted post-hoc tests indicated differences between the profiles on all ICM constructs at follow-up, with the exception of social modeling. Non-acceptingly aware participants scored significantly higher than moderate mindful eaters and acceptingly unaware participants on most ICM constructs, but lower on cognizance constructs and self-efficacy beliefs. Table 3 presents these differences between the profiles on ICM variables at T1.

**Table 3.** Differences between LPA groups on ICM variables at T1.

Construct	Total		Moderate Mindful Eater (n = 271)		Acceptingly Unaware (n = 130)		Non-Acceptingly Aware (n = 109)		F(df = 2, 510)	*p*-value	Eta-squared	Group differences (Tukey HSD)
	*M*	*SD*	*M*	*SD*	*M*	*SD*	*M*	*SD*				
Knowledge	.50	.32	.52	.31	.33	.30	.64	.28	36.18	<.001	.124	NAA **>** MME **>** AU
Susceptibility	2.74	.82	2.63	.70	2.34	.73	3.51	.71	88.18	<.001	.257	NAA **>** MME **>** AU
Severity	3.20	.77	3.17	.71	2.86	.83	3.66	.63	36.16	<.001	.124	NAA **>** MME **>** AU
Nutrition Cognizance	3.72	.78	3.80	.75	3.76	.86	3.50	.71	5.79	.003	.022	NAA < MME
Quality Cognizance	4.11	.82	4.18	.80	4.14	.95	3.89	.67	5.02	.007	.019	NAA < MME
Focus Cognizance	2.52	.67	2.53	.65	2.26	.60	2.83	.67	24.31	<.001	.087	NAA **>** MME **>** AU
Cues	2.47	1.10	2.47	1.00	1.95	.93	3.08	1.20	36.97	<.001	.127	NAA **>** MME **>** AU
Attitude Pros	4.49	1.21	4.57	1.07	3.81	1.35	5.09	.96	41.42	<.001	.140	NAA **>** MME **>** AU
Attitude Cons	3.28	.99	3.26	.93	3.10	1.09	3.51	.97	5.49	.004	.021	NAA > MME
Subjective Norms	1.95	1.18	1.88	1.11	1.79	1.13	2.32	1.35	7.08	<.001	.027	NAA > MME, AU
Modeling	2.02	1.16	2.06	1.16	1.84	1.14	2.14	1.18	1.95	.143	.008	-
Social Support	1.89	1.12	1.88	1.06	1.71	1.07	2.13	1.30	3.89	.021	.015	NAA > MME
Self-Efficacy	4.21	1.14	4.29	1.03	4.74	1.13	3.39	.97	50.65	<.001	.166	NAA **<** MME < AU
Intention	3.14	1.75	3.25	1.71	2.38	1.66	3.77	1.66	21.27	<.001	.077	NAA **>** MME **>** AU
Action Planning	1.91	1.06	1.91	1.00	1.41	.80	2.47	1.17	32.64	<.001	.113	NAA **>** MME **>** AU
Coping Planning	1.75	1.00	1.72	.96	1.32	.68	2.34	1.14	34.18	<.001	.118	NAA **>** MME **>** AU

Note. MME = Moderate Mindful Eater; AU = Acceptingly Unaware; NAA = Non-Acceptingly Aware.

## Discussion

This study aimed to investigate the degree of homogeneity within mindful eating skills by exploring the existence of distinct mindful eating profiles. We further compared these profiles with respect to their demographic characteristics and social-cognitive beliefs about mindful eating using the theoretical backdrop of the ICM.

Regarding the first research aim, our findings suggest the existence of distinct response profiles regarding predisposing mindful eating skills (e.g. the ability to observe the effects of external or internal factors on one's eating behavior or the ability to respond with acceptance toward one's eating behavior). The conducted LPA models pointed to three latent profiles with (i) moderate awareness and acceptance skills closely following the total mean; (ii) high awareness but lower than average acceptance skills; and (iii) low awareness but high acceptance skills at both baseline and 3-month follow-up. These predisposing differences challenge the notion of treating participants in mindful eating interventions as one homogenous group with equal baseline abilities and needs. Although most persons remained in the same group over time, 25-33% did transition to and from the group of moderate mindful eaters. This suggests that group membership may also be somewhat dynamic and open to change. Consistent with our findings, a previous Dutch study using the full FFaMES also observed movement patterns primarily to and from the moderate mindful eating group, but not between the other groups *(*Preissner et al., 2025c*)*. This suggests that the ‘acceptingly unaware’ and ‘nonacceptingly aware’ groups may be more stable, potentially requiring more targeted intervention efforts to facilitate meaningful eating behavior change. Further longitudinal research in more culturally diverse populations is needed to investigate whether similar group structures and transition dynamics exist across cultural contexts, and to tailor health promotion strategies accordingly.

Although profiles characterized by uniformly low or high scores on awareness and acceptance skills have been reported in some studies on dispositional mindfulness (e.g. Bronchain et al., 2021; Stanmyre et al., 2022), such profiles did not emerge in our sample regarding mindful eating. While a low-low profile might appear as something of a contradiction (i.e. lacking acceptance of food-related experiences while simultaneously being unaware of such experiences), this profile may be related to what earlier studies have described as *precontemplators* using the Transtheoretical Model (Levesque et al., 2000) or *immotives* using the I-Change Model (Dijkstra et al., 1998; Kremers et al., 2001). One potential explanation for the absence of this profile may be that individuals uninterested in reflecting on their eating behavior may have been less likely to participate in this study. The high-high profile, while potentially optimal for having a healthy relationship with food (Carrière et al., 2022; Carrière et al., 2022), may be more likely in populations that are experienced meditators or familiar with other mindfulness-based exercises. The absence of a distinct high-high profile may be explained by the limited familiarity with mindfulness-based exercises in our sample.

In line with our second aim, we found the three profiles to differ in socio-demographic characteristics and social-cognitive beliefs regarding mindful eating. Individuals with low awareness but high acceptance skills (‘acceptingly unaware’) were most likely to be older, male, and with a lower educational status. This group consistently scored the least favorable on pre-motivational, motivational, intentional, and planning constructs. This suggests that having high acceptance skills does not translate into an active interest in adopting mindful eating. Because this group does not seem to evaluate the physical sensations regarding eating (e.g. hunger cues, taste and smells), thoughts, emotions, and their eating behavior, they may also be less likely to experience emotional distress that could lead to cognitive and behavioral coping strategies (Siemers et al., 2025). As a result, they might not feel the need to push eating-related sensations, thoughts, and emotions away. In other words, their higher scores on non-judgmental and non-reactive skills seem to be due to them not perceiving a problem that would require ‘better’ self-regulation.

In contrast, the high awareness, low acceptance profile (‘non-acceptingly aware’) consisted mostly of higher-educated females with a higher BMI, who tended to score the highest on ICM constructs. Despite perceiving themselves as vulnerable to developing health issues and having strong intentions and plans to practice mindful eating in the future, this group also showed the lowest self-efficacy beliefs in combination with the highest perceived cons of mindful eating (e.g. ‘ … it would make me feel guilty about how I normally eat’, ‘ … I will no longer be able to eat everything I want to eat’, ‘ … it will make me feel stressed about eating’). Unlike the acceptingly unaware group, where low awareness prevents engagement, the non-acceptingly aware group is highly conscious of their eating behavior and related thoughts, emotions, and physical sensations. They might recognize the potential consequences of their eating behavior and may want to practice mindful eating, but may also be realistic about its difficulty to achieve this. Additionally, the relatively low scores on non-judgmental and non-reactive acceptance skills may also be a barrier for adopting mindful eating as confronting strongly perceived internal (e.g. guilt or shame for overeating or giving into cravings) and external experiences (e.g. exposure to environmental food cues) may feel restrictive and stressful. Hence, a challenge for this group will be how to increase their low-self-efficacy feelings, perhaps by (i) setting more realistic and feasible goals and (ii) addressing underlying emotional barriers. Future studies may want to test experimentally which approach may be beneficial for this group.

Although further research is needed to confirm these exploratory findings and interpretations, this study expands on prior research on the interplay between awareness and acceptance components (Carrière et al., 2022; Keirns et al., 2021; Lacaille et al., 2014; Mantzios & Wilson, 2015; Peitz et al., 2021). As LPA are particularly well-suited for studying multifaceted phenomena (Ilie et al., 2024), the present study identified important underlying mechanisms and their interactions not previously captured by single composite score measures of mindful eating (Preissner et al., 2022). The additional validation of the LPA results using the ICM constructs added valuable information about group distinctions in social-cognitive beliefs that may pose as barriers or facilitators of eating behavior change.

The present findings have several implications for research and practice. First, ‘nonacceptingly aware’ participants represent a high-risk group due to their high emotional reactivity and harsh judgment of themselves and their eating behavior, paired with being highly aware of internal and external cues to eat. This finding is in line with prior research suggesting that a person may be aware of the thoughts, emotions, and physical sensations that accompany their eating but have difficulty successfully maintaining a mental distance from habitual patterns of reaction to cravings and cues to eat and accompanying self-judgment (Lacaille et al., 2014; Tapper, 2017). The implications of this are already somewhat apparent in the present study, with 10% of this group disclosing that they are struggling with an eating disorder. As individuals with overweight and obesity typically show both a reduced ability to regulate emotions and a tendency to eat in response to negative emotions (Frayn & Knäuper, 2018; Geliebter & Aversa, 2003; Konttinen et al., 2010; Mantzios & Wilson, 2015; Schag et al., 2013), interventions promoting only awareness of hunger and food cues might trigger more engagement in problematic eating behaviors (Siemers et al., 2025). It is key for researchers to be aware of such differences in their intended target population and the potential inadvertent effects of purely training awareness (Siemers et al., 2025). A careful evaluation of existing mindfulness-based programs as well as other programs utilizing purely awareness training (e.g. ‘attentive eating’, ‘focused attention while eating’, and ‘focused eating’; Long et al., 2011; Whitelock et al., 2018) is warranted to ensure that such programs are safe and effective for people with heightened awareness and reduced acceptance of their food-related thoughts, emotions, and experiences.

While mindfulness-based practices aim to target awareness and acceptance of inner-individual and environmental experiences in the present-moment, they do not directly address knowledge, perceived pros and cons, and the enhancement of self-efficacy regarding routine practice of mindful eating. Therefore, additional behavior change strategies may be necessary, such as: (i) enactive mastery experiences, (ii) vicarious experiences through role models, (iii) verbal persuasion/encouragement, (iv) improving physical and emotional states, and (v) reattribution training (Bandura, 1997; Eldredge et al., 2016). The combination of mindfulness-based practices and traditional health behavior change components is not new and already included in existing programs: For example, Mindfulness-Based Cognitive Therapy (MBCT; Segal et al., 2002) incorporates action planning and Acceptance and Commitment Therapy (ACT; Hayes et al., 1999) incorporates value clarification exercises and goal setting (Tapper, 2017). As the present suggestions for the inclusion of behavior change methods extend beyond the scope of traditional mindfulness practice, their effects on behavior change must be experimentally evaluated separately from mindfulness-based practices (Tapper, 2017).

Taken together, the present findings point to the limitations of a one-size-fits-all approach to mindful eating. Researchers may need to consider different targeted intervention strategies based on baseline social-demographic characteristics and social-cognitive beliefs (Ilie et al., 2024). In addition, mindful eating research should develop and test tailored recruitment strategies to ensure diverse participants for study evaluation. Given that most participants in our study lacked prior awareness of and knowledge about mindful eating, recruitment and early intervention efforts should prioritize raising awareness about what mindful eating is and how it can benefit individuals. These efforts should focus on helping people recognize their current eating behaviors (i.e. develop behavioral cognizance), improve their knowledge of what mindful eating entails (i.e. the suggested benefits of acceptance), and highlight the risks associated with dysregulated eating behaviors. Additionally, interventions should emphasize the personal benefits of mindful eating, e.g. better control over eating habits. Practical strategies could include using informational campaigns, interactive workshops, and more targeted messaging that connects mindful eating to participants’ individual health concerns and goals (Preissner et al., 2022). However, further longitudinal research is needed to select the most relevant beliefs and constructs to target in interventions.

### Strengths and limitations

To our knowledge, this is the first prospective study to investigate mindful eating skills in combination with theory-based determinants of mindful eating. A validated scale was utilized to study group differences in pre-disposing awareness and acceptance skills, with the latter commonly being disregarded by other mindful eating measures (Carrière et al., 2022; Mantzios, 2021). The chosen LPA approach is novel to mindful eating as it moves beyond the assumptions of population homogeneity (Collins & Lanza, 2009) and offers valuable insight for future targeting and optimizing of mindful eating research. Nonetheless, we acknowledge the following limitations.

First, the three-profile solution identified through the LPA models constitutes one possible solution that emerged through the exploration of multiple models and fit indices. As LPA relies on a combination of various fit indices and theoretical considerations (Wang & Hanges, 2011) as well as the plausibility and interpretability of groups (Spurk et al., 2020), the present number of three profiles is not fixed and may vary across different populations, in intervention contexts, as well as over longer follow-up periods. Future research could, for example, conduct (i) longitudinal transition analyses to examine whether participants remain or transition between profiles over the course of an intervention and (ii) LPA on social-cognitive beliefs to identify other meaningful respondent subgroups in beliefs (see, e.g. Beattie et al., 2022).

A second limitation is that, although the FFaMES considers both awareness and acceptance components, its non-reactivity subscale has previously been criticized for resembling the construct of emotional eating (Mantzios, 2023a). Such conceptual ambiguity may have reduced the precision of profile identification. To improve consistency in mindful eating research and practice, Mantzios (2023b) recently proposed a distinction between decision-making for mindful eating and mindful eating behavior alongside a measure for mindful eating behavior. As these recommendations for separation were published after the date of obtaining ethical approval for this study, we were unable to consider this distinction in mindful decision-making and mindful eating behavior in the present study. Nonetheless, we acknowledge and agree with the importance of this distinction in definition and utilizing corresponding measurement in future research. We similarly acknowledge the potential conceptual overlap of quality cognizance items (i.e. the subjective perception of engaging in behaviors related to mindful eating) with those of the awareness subscales of the FFaMES (e.g. how frequently an individual is aware of thoughts or emotions before/during/after eating) due to the shared topic of mindful eating. Future research is needed to refine the measurement of behavioral cognizance and ensure its distinction from mindfulness-related constructs.

Third, we noticed between 17 and 18% participant dropout per time point similar to other longitudinal research using online panels (Roberts et al., 2022). Dropouts were significantly younger than completers, suggesting that individuals more likely to experience changes in eating behavior due to shifting routines and environments were underrepresented in the final sample.

## Conclusion

Established theoretical models, such as the I-Change Model, in combination with latent profile analysis, can help identify and target groups of individuals with different socio-demographic and behavioral characteristics. Our findings highlight three groups with distinct patterns of mindful eating skills using a multidimensional measure. As these groups differed in demographic and social-cognitive beliefs, the present results suggest a need for more targeted recruitment and interventions. For example, individuals low in awareness may benefit from early-stage intervention components focused on improving behavioral cognizance and highlighting the personal relevance of mindful eating, whereas those with higher awareness but lower acceptance may require greater emphasis on emotion regulation strategies and raising self-efficacy. Targeting mindful eating interventions to the identified profiles as well as pre-motivational, motivational, intentional, and planning constructs ensures that different groups of individuals are represented in and can benefit from interventions in a safe and accessible way. Screening participants at baseline using brief questionnaires assessing mindful eating skills could be a practical step that allows practitioners to adapt entry points, differently cultivate awareness and acceptance skills (or their combination), and to select behavioral strategies (e.g. building self-efficacy) appropriate to each subgroup. Longitudinal, experimental research is necessary to replicate the present findings and identify the most relevant and effective determinants of behavior adoption, change, and maintenance using more refined measures of mindful eating.

## Author contributions

CP and HdV contributed to the study's conception and design. Analyses were performed by CP. The first draft of the manuscript was written by CP and all authors were involved in revisions of the previous versions of the manuscript. All authors read and approved the final manuscript.

## Figures and Tables

**Figure A1.**
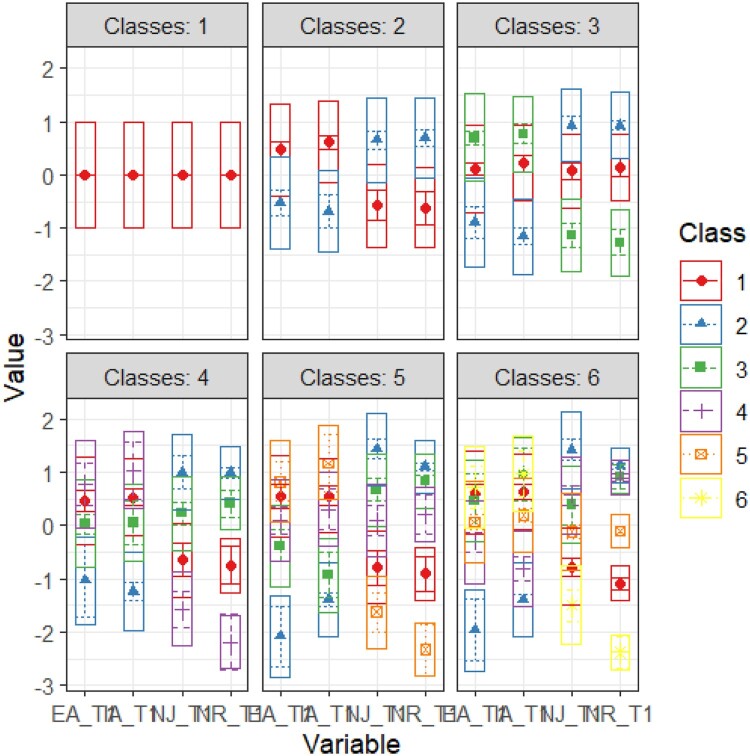
Plots depicting different model solutions (Model 1) for FFaMES facets at baseline (*n* = 613). Note. FFaMES facets were renamed to end in _T1 instead of _T0 as a result of R returning errors in other, unrelated data reshaping attempts. EA = External Awareness, IA = Internal Awareness, NJ = Non-Judgment, NR = Non-Reactance.

**Figure A2.**
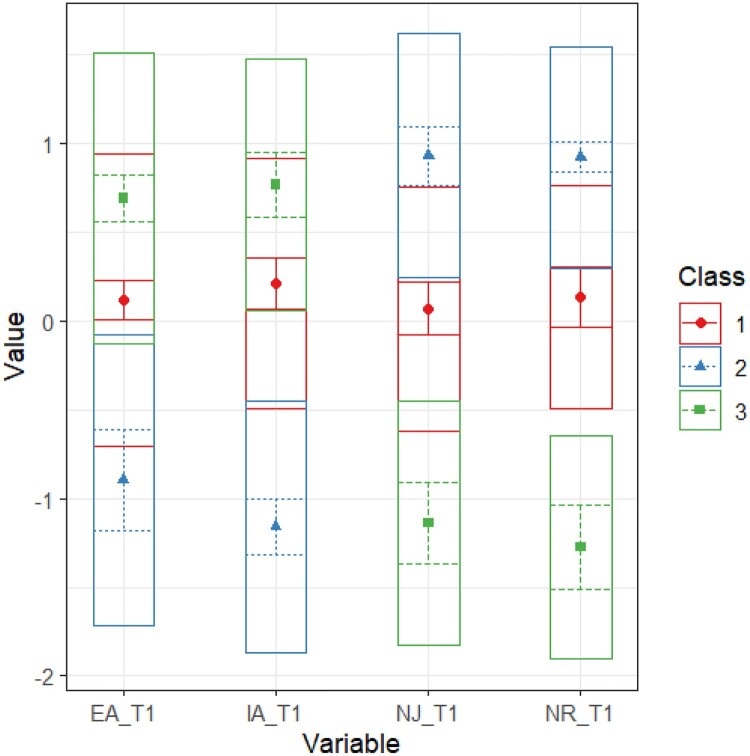
Plots of the extracted three-profile LPA model for FFaMES facets at baseline (*n* = 613). Note. FFaMES facets were renamed to end in _T1 instead of _T0 as a result of R returning errors in other, unrelated data reshaping attempts. EA = External Awareness, IA = Internal Awareness, NJ = Non-Judgment, NR = Non-Reactance.

**Figure A3.**
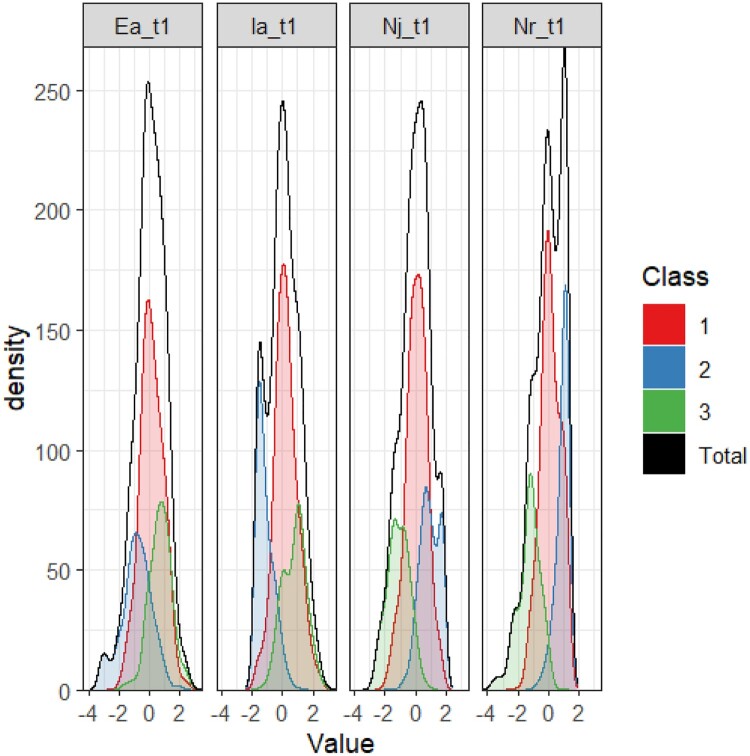
Density plots of the extracted three-profile LPA model for FFaMES facets at baseline (*n* = 613). Note. FFaMES facets were renamed to end in _T1 instead of _T0 as a result of R returning errors in other, unrelated data reshaping attempts. EA = External Awareness, IA = Internal Awareness, NJ = Non-Judgment, NR = Non-Reactance.

**Figure A4.**
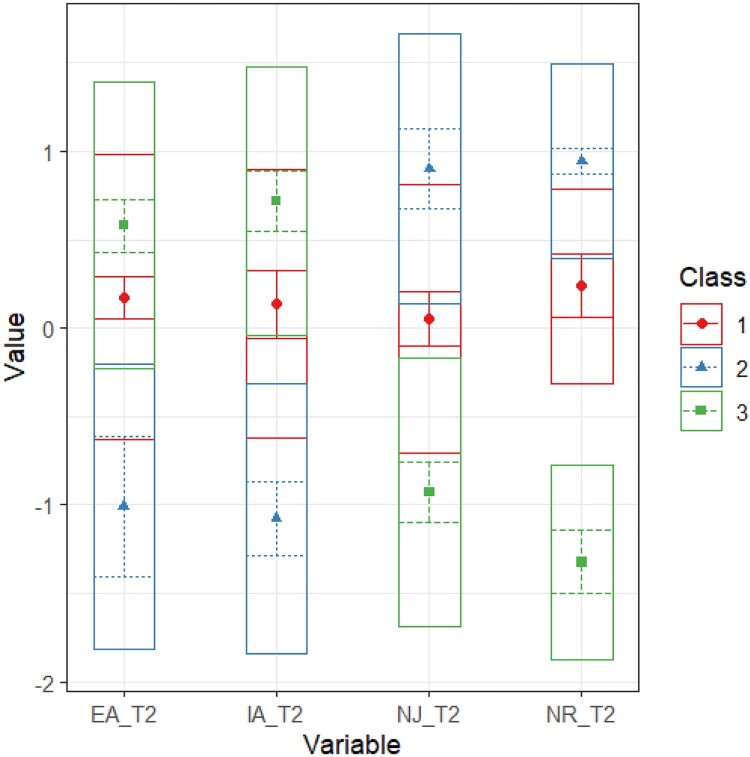
Plots of the extracted three-profile LPA model for FFaMES facets at follow-up (*n* = 511). Note. FFaMES facets were renamed to end in _T2 instead of _T1 as a result of R returning errors in other, unrelated data reshaping attempts. EA = External Awareness, IA = Internal Awareness, NJ = Non-Judgment, NR = Non-Reactance.

## Data Availability

The dataset underlying the current study is openly available in OSF upon publication (osf.io/9ct6j).

## Open Scholarship

This article has earned the Center for Open Science badges for Open Data, Open Materials and Preregistered+. The data and materials are openly accessible at osf.io/u9pkv.

## Appendix A

### Output LPA models and plots at baseline

**Table A1.** R Output LPA with equal variances and 0 covariance at T0 (*n* = 613).

Model	Classes	AIC	BIC	Entropy	prob_min	prob_max	n_min	n_max	BLRT_p
1	1	5704.83	5740.17	1.00	1.00	1.00	1.00	1.00	
1	2	5177.96	5235.38	0.72	0.91	0.92	0.48	0.52	0.01
1	3	4967.88	5047.38	0.75	0.87	0.90	0.22	0.53	0.01
1	4	4909.35	5010.94	0.76	0.84	0.87	0.06	0.42	0.01
1	5	4879.42	5003.09	0.77	0.67	0.89	0.06	0.40	0.01
1	6	4792.29	4938.05	0.84	0.73	0.94	0.05	0.35	0.01
1	7	4792.76	4960.60	0.80	0.62	0.94	0.04	0.35	0.17
1	8	4775.61	4965.52	0.75	0.63	0.96	0.04	0.21	0.01
1	9	4774.86	4986.87	0.77	0.53	0.95	0.02	0.27	0.11

Note. AIC = Akaike information criterion; BIC = Bayesian information criterion; n_min = proportions of the sample assigned to the smallest class; n_max = proportions of the sample assigned to the largest class; BLRT_p = p-value of the bootstrapped likelihood ratio test.

**Table A2.** R Output LPA general model with standardized variables at T0 (*n* = 613).

Model	Classes	AIC	BIC	Entropy	prob_min	prob_max	n_min	n_max	BLRT_p
1	1	6959.12	6994.45	1.00	1.00	1.00	1.00	1.00	
1	2	6432.31	6489.73	0.72	0.91	0.92	0.48	0.52	0.01
1	3	6222.19	6301.69	0.75	0.86	0.90	0.24	0.52	0.01
1	4	6163.81	6265.39	0.76	0.84	0.87	0.06	0.42	0.01
1	5	6123.46	6247.13	0.75	0.73	0.86	0.04	0.41	0.01
1	6	6046.65	6192.40	0.84	0.72	0.94	0.05	0.35	0.01

Note. AIC = Akaike information criterion; BIC = Bayesian information criterion; n_min = proportions of the sample assigned to the smallest class; n_max = proportions of the sample assigned to the largest class; BLRT_p = p-value of the bootstrapped likelihood ratio test.

**Table A3.** R Output LPA various specifications of variance and covariance at T0 (*n* = 613).

Model	Classes	AIC	BIC	Entropy	prob_min	prob_max	n_min	n_max	BLRT_p
2	1	5704.83	5740.17	1.00	1.00	1.00	1.00	1.00	
2	2	5003.30	5078.38	0.88	0.93	0.98	0.26	0.74	0.01
2	3	-							
2	4	-							
2	5	-							
2	6	-							
3	1	4961.95	5023.79	1.00	1.00	1.00	1.00	1.00	
3	2	4941.28	5025.20	0.84	0.55	0.99	0.05	0.95	0.01
3	3	4889.20	4995.21	0.74	0.60	0.94	0.05	0.74	0.01
3	4	4844.22	4972.31	0.77	0.70	0.91	0.07	0.46	0.01
3	5	4818.25	4968.42	0.77	0.69	0.91	0.05	0.46	0.01
3	6	4807.66	4979.91	0.73	0.63	0.86	0.05	0.36	0.03
6	1	4961.95	5023.79	1.00	1.00	1.00	1.00	1.00	
6	2	-							
6	3	-							
6	4	-							
6	5	-							
6	6	-							

Note. AIC = Akaike information criterion; BIC = Bayesian information criterion; n_min = proportions of the sample assigned to the smallest class; n_max = proportions of the sample assigned to the largest class; BLRT_p = p-value of the bootstrapped likelihood ratio test. Model numbers correspond to Rosenberg (2021) and Rosenberg et al., (2018).

### Output LPA models and plots at follow-up

**Table A4.** R Output LPA various specifications of variance and covariance at T0 (*n* = 511).

Model	Classes	AIC	BIC	Entropy	prob_min	prob_max	n_min	n_max	BLRT_p
1	1	4720.31	4754.19	1.00	1.00	1.00	1.00	1.00	
1	2	4322.98	4378.03	0.76	0.89	0.96	0.35	0.65	0.01
1	3	4157.80	4234.02	0.77	0.85	0.91	0.23	0.52	0.01
1	4	4118.14	4215.53	0.77	0.71	0.91	0.08	0.45	0.01
1	5	4082.46	4201.03	0.77	0.72	0.91	0.05	0.37	0.01
1	6	3986.09	4125.82	0.89	0.75	0.97	0.04	0.35	0.01
2	1	4720.31	4754.19	1.00	1.00	1.00	1.00	1.00	
2	2	4199.59	4271.58	0.86	0.92	0.97	0.26	0.74	0.01
2	3	4012.47	4122.57	0.81	0.89	0.94	0.17	0.53	0.01
2	4	3978.51	4126.71	0.80	0.72	0.95	0.06	0.45	0.01
2	5	-							
2	6	-							
3	1	4161.08	4220.36	1.00	1.00	1.00	1.00	1.00	
3	2	4104.42	4184.88	0.77	0.87	0.96	0.26	0.74	0.01
3	3	4085.38	4187.00	0.77	0.62	0.95	0.05	0.69	0.01
3	4	4061.25	4184.05	0.75	0.65	0.93	0.05	0.62	0.01
3	5	4063.37	4207.34	0.66	0.56	0.89	0.05	0.50	0.29
3	6	4044.12	4209.26	0.73	0.51	0.94	0.05	0.38	0.01
6	1	4161.08	4220.36	1.00	1.00	1.00	1.00	1.00	
6	2	-							
6	3	-							
6	4	-							
6	5	-							
6	6	-							

Note. AIC = Akaike information criterion; BIC = Bayesian information criterion; n_min = proportions of the sample assigned to the smallest class; n_max = proportions of the sample assigned to the largest class; BLRT_p = p-value of the bootstrapped likelihood ratio test. Model numbers correspond to Rosenberg (2021) and Rosenberg et al., (2018).

**Table A5.** R Output LPA general model with standardized variables at T0 (*n* = 511).

Model	Classes	AIC	BIC	Entropy	prob_min	prob_max	n_min	n_max	BLRT_p
1	1	5801.27	5835.14	1.00	1.00	1.00	1.00	1.00	
1	2	5404.01	5459.06	0.75	0.87	0.96	0.36	0.64	0.01
1	3	5238.68	5314.89	0.77	0.86	0.90	0.23	0.51	0.01
1	4	5199.11	5296.50	0.77	0.73	0.91	0.08	0.45	0.01
1	5	5163.51	5282.07	0.77	0.72	0.91	0.05	0.37	0.01
1	6	5166.57	5306.30	0.70	0.40	0.90	0.04	0.35	0.29

Note. AIC = Akaike information criterion; BIC = Bayesian information criterion; n_min = proportions of the sample assigned to the smallest class; n_max = proportions of the sample assigned to the largest class; BLRT_p = p-value of the bootstrapped likelihood ratio test.
